# Modeling the filtration efficiency of a woven fabric: The role of multiple lengthscales

**DOI:** 10.1063/5.0074229

**Published:** 2022-03-01

**Authors:** Ioatzin Rios de Anda, Jake W. Wilkins, Joshua F. Robinson, C. Patrick Royall, Richard P. Sear

**Affiliations:** 1 H. H. Wills Physics Laboratory, University of Bristol, Tyndall Avenue, Bristol BS8 1TL, United Kingdom; 2 School of Mathematics, University of Bristol, University Walk, Bristol BS8 1TW, United Kingdom; 3 Department of Physics, University of Surrey, Guildford GU2 7XH, United Kingdom; 4 Institut für Physik, Johannes Gutenberg-Universität Mainz, Staudingerweg 7-9, 55128 Mainz, Germany; 5 Gulliver UMR CNRS 7083, ESPCI Paris, Université PSL, 75005 Paris, France; 6 School of Chemistry, University of Bristol, Cantock's Close, Bristol, BS8 1TS, UK

## Abstract

During the COVID-19 pandemic, many millions have worn masks made of woven fabric to reduce the risk of transmission of COVID-19. Masks are essentially air filters worn on the face that should filter out as many of the dangerous particles as possible. Here, the dangerous particles are the droplets containing the virus that are exhaled by an infected person. Woven fabric is unlike the material used in standard air filters. Woven fabric consists of fibers twisted together into yarns that are then woven into fabric. There are, therefore, two lengthscales: the diameters of (i) the fiber and (ii) the yarn. Standard air filters have only (i). To understand how woven fabrics filter, we have used confocal microscopy to take three-dimensional images of woven fabric. We then used the image to perform lattice Boltzmann simulations of the air flow through fabric. With this flow field, we calculated the filtration efficiency for particles a micrometer and larger in diameter. In agreement with experimental measurements by others, we found that for particles in this size range, the filtration efficiency is low. For particles with a diameter of 1.5 *μ*m, our estimated efficiency is in the range 2.5%–10%. The low efficiency is due to most of the air flow being channeled through relatively large (tens of micrometers across) inter-yarn pores. So, we conclude that due to the hierarchical structure of woven fabrics, they are expected to filter poorly.

## I. INTRODUCTION

During the COVID-19 (Corona Virus Infectious Disease 2019) pandemic, billions of people have worn masks (face coverings) to protect both themselves and others from infection.^1–5^ There are three basic types of mask or face covering. Surgical masks and respirators are made of non-woven materials, while cloth masks are made of woven material. Filtration of air by non-woven materials is well studied.^6^ However, pre-pandemic, very little research was done into filtration by woven materials, which have a different structure to that of non-woven materials. Here, we try and address this, by studying how a woven fabric filters small particles out of the air.

**FIG. 1.**
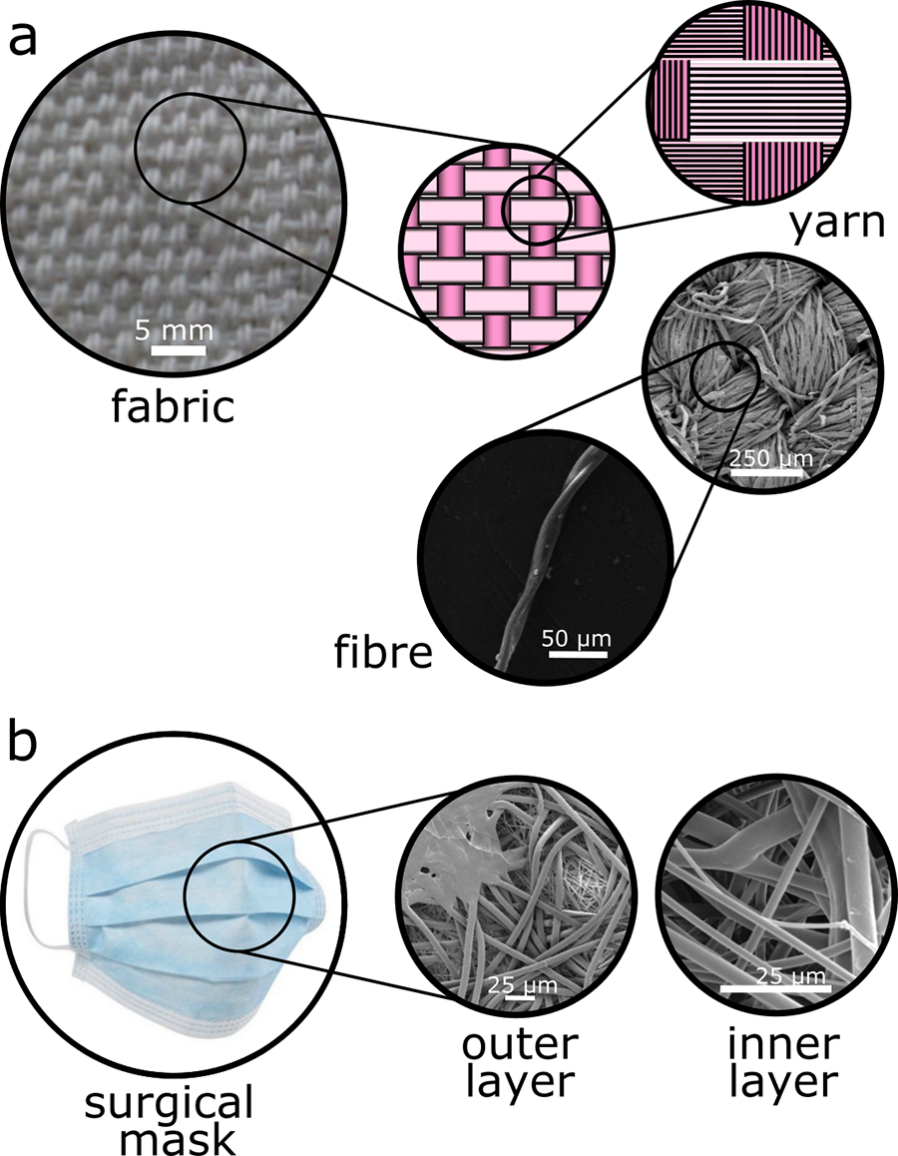
(a) Fabric is a porous material with structure on multiple lengthscales. For the top three images, from left to right we look at successively smaller lengthscales. At the largest lengthscale, the fabric is a lattice woven from perpendicular yarns that go over and under other yarns at right angles to them. In the middle schematic, vertical yarns are shown as dark pink, horizontal yarns as pale pink. As illustrated in both the top right schematic and the SEM images on the right, these yarns are made by twisting together many, much smaller fibers. At the bottom of figure (a), we show a single fiber. Fibers are of order 10 *μ*m in diameter while yarns are a few hundred *μ*m across. (b) From left to right, we have an image of a typical surgical mask, and SEM images of the fibers of which it is made. Note that the fibers are randomly distributed, there is no lengthscale above that of the fibers, and the fibers in a filtering inner layer of a surgical masks typically have diameters a little less than 10 *μ*m, Lee *et al.*^11^ quote a mean diameter of 5.5 *μ*m.

Woven fabrics have a very different structure from surgical masks. We compare the structures of woven fabrics and surgical masks in Fig. 1. Surgical masks are meshes of long, thin fibers,^6^ with diameters of a few micrometers to ten micrometers, see Fig. 1(b). However, fabrics are different; they are woven from cotton (or polyester, silk, etc.) yarn. Cotton yarn is a few hundred micrometers thick, and is composed of cotton fibers, each of an order of ten micrometers thick. These fibers are twisted into yarns, which are, in turn, woven into the fabric,^7^ see Fig. 1. This two-lengthscale (fiber and yarn) hierarchical structure of fabrics is known to affect the fluid flow through them, which has been studied in the context of laundry.^8,9^ However, there has been little effort to study its effect in the context of particle filtration.^10^

To understand how woven fabrics filter air, we started by using a confocal microscope to obtain a three-dimensional image of a sample of fabric, at a spatial sampling rate of 1.8 *μ*m. This image is then used as input to lattice Boltzmann (LB) simulations of air flow inside a woven face mask during breathing. That flow field is then used to calculate large numbers of particle trajectories through the fabric to estimate filtration efficiencies.

### A. Previous work on filtration by woven fabrics

Konda *et al.*,^12,13^ Duncan *et al.*,^14^ and Sankhyan *et al.*^15^ have all measured filtration efficiencies for a number of fabrics. They studied the filtration of particles in the size range we consider, which is ≥1 *μ*m. Zangmeister *et al.*^16^ studied the mechanism of filtration for smaller particles. Note that the original measurements of Konda and co-workers suffered from methodological problems,^13,17–19^ which were later corrected.^13^

This work directly measured filtration efficiencies but did not image the fabric in three dimensions. Lee *et al.*,^20^ Du *et al.*,^21^ and Lee *et al.*^11^ imaged the filtration media of surgical masks,^20,21^ or of respirators with the surface charges removed, making the filtration media similar to that of many surgical masks.^11^ However, Lee *et al.*^20^ and Du *et al.*^21^ did not use these imaging data to compute filtration efficiencies, while Lee *et al.*^11^ only performed relatively limited studies of filtration efficiency.

### B. Evidence that droplets approximately a micrometer in diameter carry infectious SARS-CoV-2 virus

The literature on COVID-19 transmission is large but it is worth briefly summarizing the part most relevant to this work. The breath we exhale is an aerosol of small mucus droplets in air that is warm and humid because it has come from our lungs.^22^ These droplets range in size from much less than a micrometer to hundreds of micrometers.^23^ Vocalization (i.e., speech or singing) produces more aerosol than ordinary breathing.^23–25^ The peak in the size distribution function of exhaled droplets is around 1.6 *μ*m—this is the count median diameter of Johnson and co-workers.^23^

The median diameter of 1.6 *μ*m is for droplets as exhaled in our breath, breath which is essentially saturated with water vapor, i.e., at essentially 100% relative humidity (RH).^22^ It takes only a few milliseconds for droplets to pass through a mask filter (see Sec. VII) and this short time combined with the 100% RH means that droplets do not evaporate while passing out through a mask filter. If a person inhales another person's breath more-or-less directly, for example if they are close and talking to each other, then the droplets inhaled will not have left the humid breath, and still have the same diameter as when they were exhaled.

However, when our breath mixes with room air,^22,26–28^ the humidity drops. Then, micrometer-sized droplets evaporate in timescales of order 10 ms.^29^ After this evaporation, the droplet diameter is smaller by a factor of 2 to 3.^23,29,30^ So, typical droplet sizes are around 1.6 *μ*m as we breathe them out through a mask, but around 0.5–0.8 *μ*m when we breathe them in. We do not expect droplets to pick up significant amounts of water on inhalation through a filter, as the droplets will be in air from the surroundings, and they spend only a few milliseconds passing through the filter.

Both 1.6 *μ*m and around 0.5–0.8 *μ*m are approximate (count) medians of broad distributions.^23^ Due to this evaporation after exhalation, there are two sets of droplet size distributions to consider when studying filtration, with the distribution on exhalation being two to three times larger in diameter than on inhalation. The particles that need to be filtered for source control are larger than those needed to be filtered to protect the wearer.

Coleman and co-workers^31^ found SARS-CoV-2 viral RNA in both particles with diameters smaller than and larger than 5 *μ*m, and found that most of the viral RNA was in droplets with diameters less than 5 *μ*m. These correspond to diameters after evaporation. Santarpia and co-workers^32^ found infectious virus in particles both with diameters < 1 *μ*m and in the range 1–4 *μ*m, but not in particles larger than 4.1 *μ*m. Hawks and co-workers^33^ were also able to obtain infectious virus in aerosols smaller than 8 *μ*m. It should be noted that the study of Hawks and co-workers was of infected hamsters, not humans. Finally, Dabisch and co-workers infected macaques with an aerosol of droplets with median diameter 1.4 *μ*m.^34^ This body of very recent work suggests that aerosol particles of order a micrometer carry most of the virus.

It is also worth noting that Coleman and co-workers^31^ also found that the amount of viral RNA varied widely from one person to another. Some infected people breathed out no measurable RNA. Those that did breathed out an amount that varied by a factor of almost a hundred. Viral RNA was found even for those who never developed COVID-19 symptoms, i.e., who always remained asymptomatic.

As we state above, we use the term “droplet” to cover all sizes from much less than a micrometer to hundreds of micrometers and more. This is in line with the aerosol and fluid mechanics literature, but some works in the medical literature reserve the term “droplet” for diameters over 5 *μ*m, despite there being no justification for this distinction.^35,36^

### C. Evidence that masks filter out SARS-CoV-2

Adenaiye and co-workers^37^ studied the effect of masks on the amount of viral SARS-CoV-2 RNA breathed out. This study tested a wide range of masks as the participants were asked to bring their own masks. They found that in “fine aerosols (<5 *μ*m),” masks reduced the amount of viral RNA detected by 48% (95% confidence interval 3%–72%), while for larger aerosols, masks reduced the viral RNA by 77% (95% confidence interval 51%–89%). Here, 5 *μ*m is presumably the evaporated diameter (not radius) but this was not specified by the authors.

### D. Mechanism of filtration

Filtration is traditionally ascribed to a sum of four mechanisms,^6^ the idea being that a particle with zero size, zero inertia, zero diffusion, and zero charge will follow the streamlines perfectly and not be filtered out. However, deviations from any one of those four conditions can cause a collision and hence filtration.

The four mechanisms are as follows:

1. *Interception:* Particles whose center of mass follows streamlines perfectly can still collide with fibers, if the particles have a non-zero size. This is a purely geometric mechanism that does not require inertia.
2. *Inertial:* With inertia, particles cannot follow the air streamlines perfectly. While a streamline goes around an obstacle, a particle with inertia will deviate from the streamline and so may collide.
3. *Diffusion:* Particles diffuse in air, creating further deviations from streamlines and thus potential collisions with the obstacle.
4. *Electrostatic interactions:* Charges, dipole moments, etc., on the fibers and on the droplets will interact with each other. If they pull the two toward each other, this will enhance filtration. Cotton fibers have no charge distribution as far as we know, so we do not expect this to be a significant mechanism here.

Note that in practice, these mechanisms are never completely independent.^6^

Flow through masks is sufficiently slow, and the lengthscales are sufficiently small that the flow is close to Stokes flow, i.e., the Reynolds number is small. This means that streamlines do not depend on the flow speed/pressure difference. In turn, this implies that interception filtration is independent of the flow speed. Inertial filtration becomes more important with increasing flow speeds, as the faster moving particles have more inertia. While diffusion filtration becomes less efficient at faster flow speeds, as then particles spend shorter times passing through the mask. The particles then have less time to diffuse into the material of the mask, and be filtered out.

Here, we will focus on particles a micrometer or larger in size, where diffusion is less important as a filtration mechanism because particles this large diffuse slowly. So, we will focus on interception and inertial filtration. However, in the Conclusion we will return to filtration by diffusion and argue that filtration by diffusion in our fabric should be very inefficient.

The remainder of this paper is laid out as follows: Sec. II describes how we imaged the fabric and analyzed the imaging data. Section III describes our lattice Boltzmann (LB) simulations of air flow through the mask. Section IV characterizes this air flow. Sections V and VI discuss our method for calculating particle trajectories and our results for filtration, respectively. Section VII briefly discusses filtration via diffusion. Section VIII presents our conclusions.

## II. IMAGE ACQUISITION AND ANALYSIS OF A SAMPLE OF WOVEN FABRIC

In order to study filtration by woven fabrics, a high-resolution 3D image of the fabric is needed. We used confocal optical imaging to obtain an image of the fabric, at a voxel size of 1.8 *μ*m. Recent work by Lee *et al.*^20^ and by Du *et al.*^21^ has used x-ray tomography to obtain 3D images of the internal structure of surgical masks; but, to our knowledge, nobody has been able to image woven fabrics or to use confocal microscopy for this purpose, before.

The fabric was obtained from a commercial fabric mask. Square pieces of 1, 2.25, and $4\;{\text{cm}}^{2}$ were weighed individually, giving a mass per unit area of $120\;{g\;m}^{-2}$, see Table I. Using brightfield optical microscopy (Leica DMI3000 B) with a Leica 4× objective, we estimated the thickness of the fabric in air to be 285± 24 *μ*m, which we determined through different measurements along the fabric. Using the mass density of cotton, *ρ_c_*, from Table II, this corresponds to the fabric being on average about 28% cotton fibers and 72% air.

**TABLE I.** Measurements of the mass of samples of the fabric, used to determine its mass per unit area.

Area of sample ( $\;{\text{cm}}^{2}$)	Mass (g)	Mass/area (g cm^−2^)
1	0.012 10	0.012 10
2.25	0.027 42	0.012 19
4	0.048 13	0.012 03

**TABLE II.** Parameter values for masks, air, water, and mucus—all at 20 °C and atmospheric pressure 10^5^ Pa. Note that small droplets dry rapidly and this will cause their viscosity to increase. Flow rates are determined from the volume typically exhaled during one minute. Moderate exertion is defined as that readily able to be sustained daily during 8 h of work, whereas maximal exertion is the upper limit of what can be sustained for short periods of time (e.g., during competitive sports). Flow speeds are calculated for the stated mask area and flow rates assuming perfect face seal.

Quantity	Value	Reference
Air		
Mass density	$1.2\;{\text{kg}\;m}^{-3}$	41
Dynamic viscosity *μ*	$1.8\times 10^{-5}\;\text{Pa}\;s$	41
Kinematic viscosity *ν*	$1.5\times 10^{-5}\;m^{2}s^{-1}$	41
Water/mucus		
Mass density *ρ_p_* (water)	$998\;{\text{kg}\;m}^{-1}$	41
Dynamic viscosity (mucus)	0.1 Pa s	42
Mucus/air surface tension *γ*	$0.05\;{N\;m}^{-1}$	42
Cotton fibers		
Mass density *ρ_c_*	$1500\;{\text{kg}\;m}^{-3}$	43
Typical breathing flow rates		
Tidal breathing at rest	$6\;{l\;\text{min}}^{-1}$	44
During mild exertion	$20\;{l\;\text{min}}^{-1}$	44
During moderate exertion	$30\;{l\;\text{min}}^{-1}$	44
During maximal exertion	$85\;{l\;\text{min}}^{-1}$	44
Average flow speeds		
Effective mask area	$190\;{\text{cm}}^{2}$	45
Flow speed (rest)	$0.5\;{\text{cm}\;s}^{-1}$	
Flow speed (mild)	$1.8\;{\text{cm}\;s}^{-1}$	
Flow speed (moderate)	$2.7\;{\text{cm}\;s}^{-1}$	
Flow speed (maximal)	$7.5\;{\text{cm}\;s}^{-1}$	

### A. Image acquisition

In order to study the 3D structure of the fabric, square pieces of 0.5 cm of cotton were dyed with fluorescein (Sigma Aldrich) following Baatout *et al.*^38^ The dyed cotton squares were then washed in deionized water to eliminate any dye excess and left to dry under ambient conditions for 48 h. Once dried, the fabric was re-submerged in 1,2,3,4-tetrahydronaphthalene (tetralin, Sigma Aldrich). We chose this solvent due to its refractive index being close to the index of cotton [ $\eta_{D\text{tetralin}}=1.544$ (Ref. 39) and $\eta_{D\text{cotton}}=1.56–1.59$ (Ref. 40)]. Such matching is needed to allow imaging with fluorescence confocal microscopy.

The dyed fabric samples were immersed in tetralin. They were confined in cells constructed using three coverslips on a microscope slide. Two of the coverslips acted as a spacer, and they were sealed using epoxy glue. The spacing coverslips have a height of 0.56 mm, which prevented fabric compression. A confocal laser scanning microscope, Leica TCS SP8, equipped with a white light laser, was used to study the fiber structures, using a Leica HC PL APO 20× glycerol immersion objective with a 0.75 numerical aperture and a correction ring. The excitation/emission settings used for the fluorescein dye were 488 and 500 nm, respectively. Scans of the cell in the *z* axis were acquired to analyze the fiber network in 3D, where care was taken to ensure the pixel size (1.8 *μ*m) was equal along all axes.

**FIG. 2.**
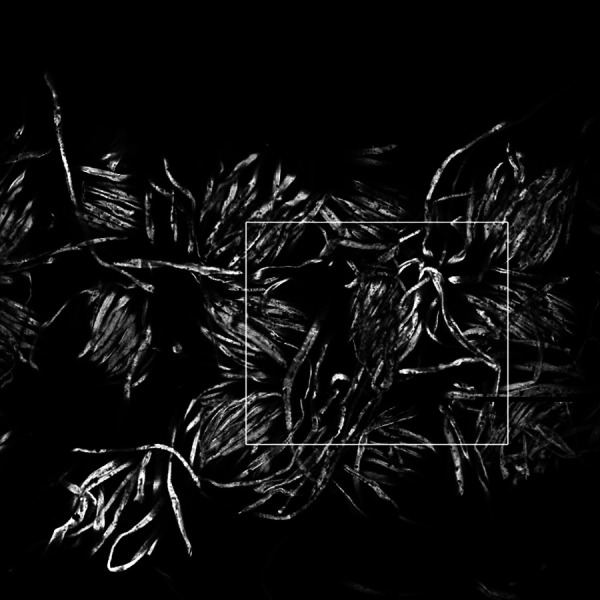
Slice (number 19, starting at 0) of the confocal image of the fabric. Slice is in the *xy* plane. The area simulated using LB is enclosed by a white box.

The confocal microscopy data are in the form of a stack of *n_z_* = 62 images of the *xy* plane, each of which is *n_x_* = 756 by *n_y_* = 756 voxels. Each voxel is a cube of side 1.8 *μ*m, see Table III. Slice number 19 (starting at zero) is shown in Fig. 2. In each slice, approximately two-thirds of the field of view is taken up with a strip of the fabric, which runs left to right in Fig. 2.

**TABLE III.** Parameter values for the fabric we have imaged, and for our lattice Boltzmann simulations. TPI is calculated by adding together number of yarns per inch along the *x* and along *y* axes.

Quantity	Value
Fabric imaged	
Cubic voxel side length	1.8 *μ*m
Total thickness imaged	62 voxels = 111.6 *μ*m
Thickness used	*L_F_* = 52 voxels = 93.6 *μ*m
Area imaged	756 × 756 voxels
	= 1 360.8 × 1 360.8 *μ*m^2^
Area used	*n_x_* = 310 to 310 + 330
	*n_y_* = 280 to 280 + 280
	= 594 × 504 *μ*m^2^
Yarn lattice constants	297 and 252 *μ*m
Threads per inch (TPI)	186
Lattice Boltzmann parameters	
Box size $n_{x}\times n_{y}\times n_{z}$	330×280×462
	= 594 × 504 × 471.6 *μ*m^3^
Darcy velocity U=Q/A	$5.6\times 10^{-7}$
Re for lengthscale 297 *μ*m	$6\times 10^{-4}$
Pressure drop	$6.7\times 10^{-6}$

Of the 62 slices, the image quality in the bottom ten is poor, due to attenuation from imperfect refractive index matching. So in effect, we can obtain good images for 52 slices, i.e., we can reliably image a section of fabric that is approximately 93.6 *μ*m thick.

### B. Fiber size distribution

**FIG. 3.**
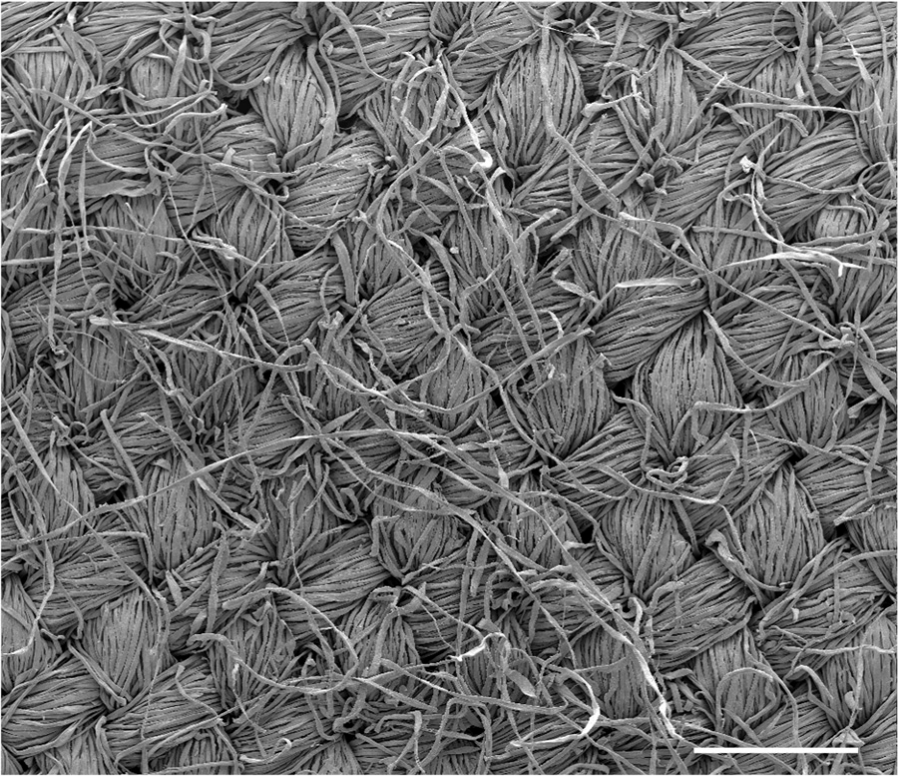
A scanning electron microscope (SEM) image of the surface of our fabric. The fabric has been coated with gold/palladium. Secondary electron images were taken at 8 kV with a 100× magnification. Scale bar = 500 *μ*m.

To obtain estimates of the distribution of fiber diameters, we imaged the surface of the fabric using a scanning electron microscope (FEI Quanta 200 FEGSEM, Thermo Fisher Scientific), see Fig. 3. We then estimated the diameter of at least 50 fibers from this image, and obtained the mean and standard deviation of fiber diameters as 16.7± 4.8 *μ*m, which we determined by analyzing the SEM images.

### C. Image analysis

The analysis of the image stack output by the confocal microscope was performed in Python using the OpenCV^46^ and cc3d^47^ packages. The confocal image stack is processed as follows:

1. We first delete the fiber voxels in the bottom ten slices due to the poorer image quality, leaving us with 52 slices of the imaged fabric. We then add 200 slices to the top, and 200 slices to the bottom, each of entirely zero intensity voxels. These additional slices are needed as the array produced for the simulations needs to cover fluid flow into and out of the fabric, i.e., we cannot just simulate flow inside the fabric, we need the approach and exit flows.
2. We then blur the image by convolving with a three-dimensional Gaussian filter that is implemented as a sequence of 1D convolution filters, with a standard deviation $\sigma_{B}=1$ voxel side (1.8 *μ*m).
3. Next, we threshold the blurred image, setting all voxels with values less than the threshold value *T* = 10 to zero, and all voxels greater than or equal to the threshold value to one. Thus we get a binary image.
4. Then, we use a 3D connected components algorithm to identify the connectivity of voxels that are one. We assign each voxel with value one to a cluster of connected voxels. All voxels of value one that are part of clusters of size *N_CL_* = 25 or less are set to zero; all other voxels of value one are assumed to be fiber voxels. N.B. Applying the Gaussian filter greatly reduces the number of connected clusters we obtain.

It is worth noting that step 4 only deletes a total of 507 voxels while keeping 11 681 929 voxels so that deleting a few isolated clusters has very little effect, and that in the final array almost 99.9% of the voxels are part of the largest cluster. This is as we should expect. Most voxels should be in a single cluster, as the fabric needs to be one connected structure in order not to fall apart.^7^ Varying the width of the Gaussian filter in the range 0.5–2 voxels has little effect. The number of voxels deleted does increase as *σ* decreases, but at σ=0.5 (and a threshold *T *=* *10) we still only delete 3099 voxels from over 11 × 10^6^, and the largest cluster has over 99.8% of the voxels.

Varying the threshold *T* (keeping *σ* = 1) in the range *T *=* *5–15 varies the number of fiber voxels by order 10%, from 13.6 × 10^6^ for *T *=* *5 to 10.2 × 10^6^ for *T *=* *15. Reducing the value of *T* makes the fibers and yarns thicker and thus the gaps in between narrower. This suggests that there is an uncertainty of about 10% in the volume of our fibers and yarns. Finally, varying the minimum cluster size *N_CL_* has little effect. Increasing it from 25 to 50 only increases the total number of fiber voxels deleted from 507 to 922, out of over 11 × 10^6^ (at *T *=* *10 and *σ* = 1).

### D. Region of the fabric studied

The fabric is essentially a rectangular lattice, woven from yarns that cross at right angles. The estimated lattice constants are given in Table III. The lattice constants are around 20 times the average fiber diameter.

**FIG. 4.**
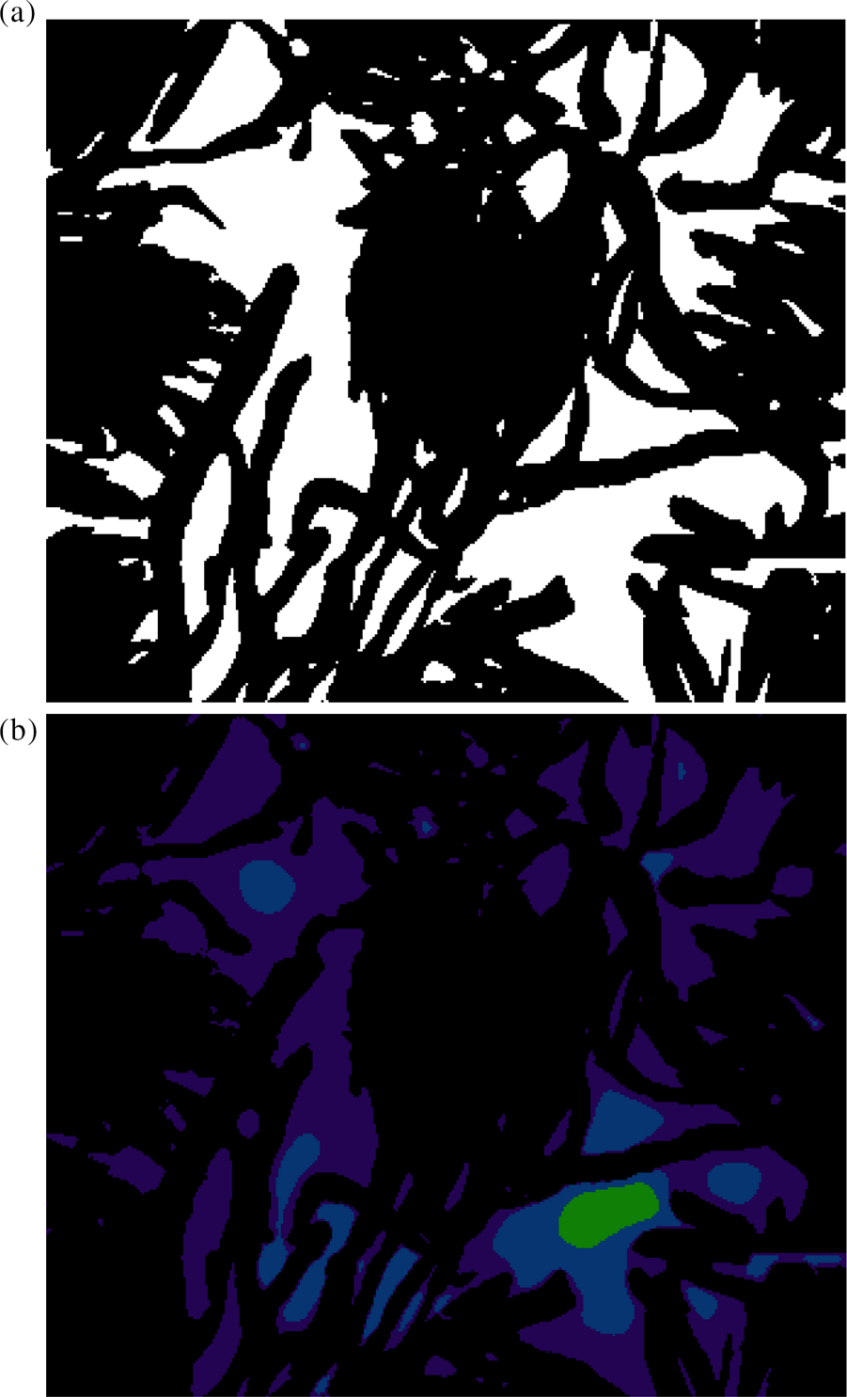
(a) The thresholded and, so, binary image produced by image analysis of the area in the white box in Fig. 2. Fiber voxels are in black and air voxels are in white. (b) Heatmap of the *z* component of velocity in the same area. Again, the black region corresponds to the fabric. The dark purple, blue, and pale green regions correspond to velocities less than the mean, between the mean and ten times the mean, and over ten times the mean velocity, respectively. The area of both images is 594 × 504 *μ*m^2^.

**FIG. 5.**
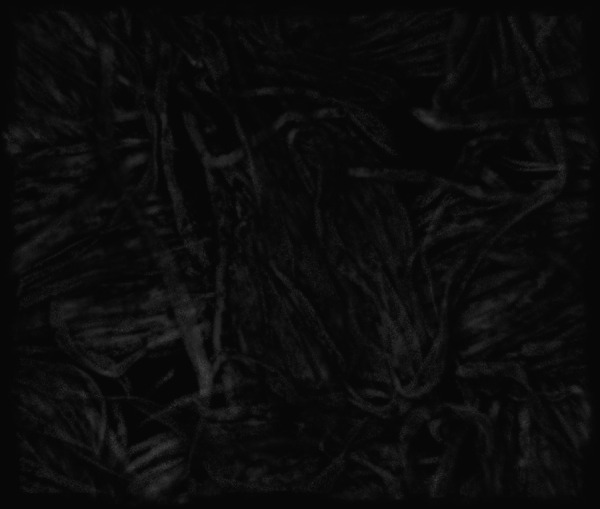
Snapshot of the movie in supplementary material that shows the part of the fabric we calculate the flow field for. Rendering done using Blender.^48^ Multimedia view: https://doi.org/10.1063/5.0074229.1
10.1063/5.0074229.1

We want to model a representative part of the fabric of a face covering, so we study an area of two by two lattice sites. This area is shown by a white box in Fig. 2, and in Fig. 4(a). Note that we put the edges of the white rectangle in the densest part of the fabric where flow is the least. The dimensions of the white rectangle are given in Table III. A full three-dimensional rendering of the region we study is shown in the supplementary material, with a snapshot in Fig. 5. The full image stack is available on Zenodo.

### E. Estimation of what fraction of the fabric thickness is in our simulation box

Using a mass density for cotton in Table II, then simply counting each voxel as (1.8 *μ*m)^3^ of cotton, we have a mass/unit area of cotton of $96\;g\;m^{-2}$ in our fabric array of 330×280×52 voxels. Our directly measured value is $120\;g\;m^{-2}$, so we estimate that our 52 slices or 93.6 *μ*m of fabric contains 80% of the mass of the fabric. However, our estimate for the fabric thickness using optical microscopy is 285 *μ*m, three times the thickness of our image.

**FIG. 6.**
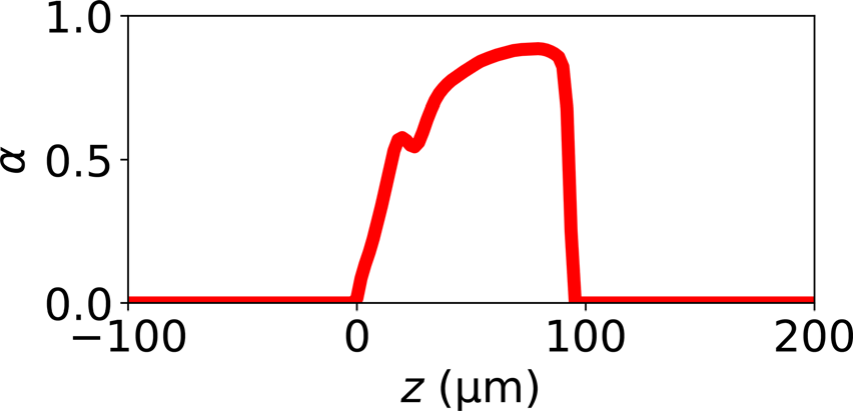
Plot of the fraction of voxels belonging to a fiber *α* (averaged over *x* and *y*), as a function of *z*. The zero of *z* is at the top of the fabric (slice 0). This is for the volume used in our simulations.

The thickness of fabric measured in air is not perfectly well defined; the fabric, being mostly air, is compressible and at the edges there are stray fibers. We have plotted the average fraction *α* of voxels that are fiber voxels, as a function of *z* in Fig. 6. Note that this is measured in the solvent. It is mostly above the average value of 28% we obtained in air, and the average value *α* inside the fabric of this plot is 69%. It is possible that the fabric may have compacted and/or the fibers swollen in our solvent.

To conclude, there is significant uncertainty in what fraction of the fabric thickness is included in the 52 slices. We can only say that our 52 slices contain at least one-third of the fabric, but probably no more than two-thirds.

## III. LATTICE BOLTZMANN SIMULATIONS OF AIR FLOW THROUGH FABRIC

Lattice Boltzmann (LB) simulations are performed on a three-dimensional lattice of *n_x_* by *n_y_* by *n_z_* lattice sites; *z* is the flow direction. We used the Palabos LB code from the University of Geneva.^49^ The code uses a standard one-relaxation-time LB algorithm on a cubic D3Q19 lattice. The speed of sound $c_{s}=1/\sqrt{3}$ in LB units where both the lattice spacing and the time step are set to one.^50^ It has a kinematic viscosity $\nu_{\text{LB}}=c_{s}^{2}\left(\omega^{-1}-1/2\right)$. We set the relaxation rate *ω* = 1 in LB units, giving a kinematic viscosity $\nu_{\text{LB}}=1/6$ in LB units.^50,51^

We run the LB simulations until the change in mean flow speed along *z* is very small so we are at steady state. We then insert particles into the resulting steady flow field to evaluate their trajectories.

Our code reads in the 330×280×462 array obtained from our image analysis. Fiber voxels have standard LB on-site bounce back^52,53^ to model stick boundary conditions for the air flow.

The box is configured such that the *x* and *y* edges are in denser parts of the fabric, so there is little flow near and at these edges. In the LB simulations, we use periodic boundary conditions (PBCs) along the *x* and *y* directions. Real fabrics are not perfectly periodic and so our flow field has artifacts near the edges. However, there is no way of avoiding artifacts at the edges, and PBCs are a simple choice.

We impose a pressure gradient along the *z* axis to drive the flow. We do this by fixing the densities in the first and last *xy* slices of the lattice along *z*. We fix the density in the *z *=* *0 slice to be $1+10^{-5}$, and that in the $z=n_{z}-1$ slice to be $1-10^{-5}$. This corresponds to a pressure difference of $(2/3)\times 10^{-5}$ across the fabric.

This small density/pressure difference across the fabric is chosen to keep the Reynolds number small, so we have Stokes flow. The Reynolds number for a flow with characteristic lengthscale *L* is

$$\text{Re}=\frac{UL}{\nu },$$ (1)

where *ν* is the kinematic viscosity and *U* is the velocity. For the velocity, we use the Darcy velocity, see Sec. IV A. The Reynolds number for the largest lengthscale (yarn lattice constant along *x*) in our simulation box is given in Table III and is much less than one; so, we have Stokes flow in our simulations.

For an air flow speed of $2.7\;{\text{cm}\;s}^{-1}$ (moderate exercise), the Reynolds number for air flow with a characteristic lengthscale of a few hundred micrometers is Re≃1. So in a fabric mask, there will be small deviations from Stokes flow, but we expect them to have little effect.

The LB simulations only give flow fields on a cubic lattice, so we use trilinear interpolation to obtain a continuous flow field $\vec{u}(\vec{r})$. Trilinear interpolation is the extension to three dimensions of linear interpolation in one dimension.^54^

## IV. AIR FLOW THROUGH THE WOVEN FABRIC

The air flow through a fabric is heavily concentrated in the inter-yarn pores, and there is essentially no flow through the centers of the yarns. This can be seen in the heatmap of the *z* velocity in Fig. 4(b). Note that all the fastest voxels (shown in pale green) are in a single patch in the middle of the biggest inter-yarn gap. There are 718 of these voxels, out of 27 190 air voxels, and they contribute over a third of the total air flow through this slice.

**FIG. 7.**
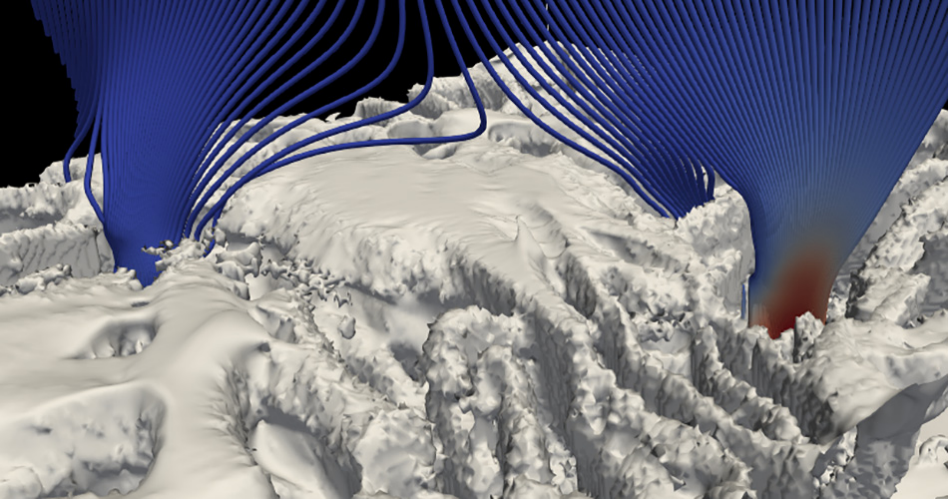
Plot of the fabric surface (white) together with streamlines. The streamlines are color coded with local velocity: blue is slow, red is fast. The flat region in the center of the image is the top of a yarn. Image produced by ParaView.^55^

The flow through the fabric is illustrated by streamlines in Fig. 7. Note that all the streamlines shown flow around the yarns and through the gaps between the yarns. We conclude that as the air goes through inter-yarn pores, the filtration efficiency will depend on whether or not particles flowing through these pores collide with the pore sides, or stray fibers across these pores.

The spacing between the fibers of a yarn is mostly too small to be resolved by our imaging technique, so presumably is mostly a micrometer or less. Note that the integrity of yarns relies on the number of physical contacts,^7^ so the fibers must touch in many places. Our limited resolution means we cannot model any flow in between the fibers. However, as the inter-yarn gaps are ∼50 *μ*m across, the flow through any gaps between fibers of order ∼1 *μ*m or less will be negligible. Assuming that flow speeds through gaps scale as one over the gap size squared, as it does in Poiseuille flow, any flow through the sub-micrometer inter-yarn gaps will be thousands of times slower than flow in the inter-yarn pores.^8,9^

Finally, the fact that the bottom-right inter-yarn pore has the largest air flow illustrates that the fabric is disordered. It is not a perfect lattice of inter-yarn pores, each of which is the same. This also means that small (in the sense of difficult to detect with the naked eye) amounts of damage to the fabric significantly affect the flow through it.

### A. Darcy's law

Fluid flow through fabrics has been studied in earlier works on the washing of fabric (laundry). The removal of dirt from fabric relies on the flow of water through it.^8,9,56,57^ These earlier works, starting with the pioneering work of van den Brekel,^8^ assumed that inter-yarn flow was dominant, which is corroborated by the present work. They modeled the flow through a fabric using the standard approach for (low Reynolds number) flow through porous media: Darcy's law.

A mask is a porous medium, and so at low Reynolds number the air flow *Q* through the fabric is given by Darcy's law^58^ as follows:

$$Q=\frac{kA}{\mu }\frac{\Delta p_{F}}{L_{F}},$$ (2)

which defines the permeability *k*. *Q* is the volume of air crossing the fabric per unit time, *A* is the area of the fabric the air flows through, and *μ* is the viscosity of air.

For our thin fabric, there are end effects. We neglect these and just consider the pressure drop across the fabric, $\Delta p_{F}$, and the thickness of the fabric, *L_F_*. The flow *Q* is proportional to the size of the pressure drop across the fabric $\Delta p_{F}$ and inversely proportional to the thickness *L_F_* of the fabric. The Darcy velocity *U* is defined by

$$U=\frac{Q}{A}.$$ (3)

In free space, *U* is the actual flow velocity, while inside a porous medium, some of the area *A* is occupied by the solid material and so does not contribute to *Q*. Then, the local flow velocity varies from point to point and is mostly higher than the Darcy velocity *U*.

In our LB simulations, we impose the pressure difference $\Delta p_{F}$ (via setting the densities at bottom and top along *z*), measure *Q*, and evaluate the permeability from

$$k=\frac{Q\mu }{A}\frac{L_{F}}{\Delta p_{F}}.$$ (4)

The viscosity of our LB fluid is $\mu =\rho_{LB}\nu_{LB}=1/6$, because $\rho_{LB}=1$ is the mass density in LB units and $\nu_{LB}=1/6$ is the kinematic viscosity also in LB units. In the same units, *L_F_* = 52.

We find a permeability of k≃0.73 in LB units, or k≃ 2.4 *μ*m^2^ on conversion using our known voxel size. This value is comparable to the value k≃ 4 *μ*m^2^ found for cotton sheets (with water as the fluid) in the experiments of van den Brekel.^8^

Note that our fabric is imaged in liquid and van den Brekel's measurements are for fabric immersed in a liquid. So it is possible that in both cases, the cotton may have swelled due to absorbing the liquid, reducing *k*. We imaged the masks in SEM (under vacuum) before and after immersion in tetralin for confocal imaging and observed no change. While, of course, it is possible that swelling occurred *during* immersion in the said solvent, we find no evidence for irreversible change due to immersion in tetralin.

### B. Impedance and pressure drop across fabric

The pressure drop across a mask must be low enough to allow easy breathing through the mask. As we have Stokes flow, the pressure drop is linearly proportional to the flow velocity, and the proportionality constant defines the mask's impedance *I,*^19^ i.e.,

$$\Delta p_{F}=IU.$$ (5)

Using Eqs. (2) and (3), we have

$$I=\mu L_{F}/k.$$ (6)

Using the viscosity of air and our estimated *k*, $I=7.1\;{\text{Pa}\;s\;\text{cm}}^{-1}$. This is of the same order as Hancock *et al.*^19^ found for 300 threads per inch (TPI) cotton. Konda *et al.*^13^ found an impedance of $4.2\;{\text{Pa}\;s\;\text{cm}}^{-1}$ for a 180 TPI cotton/polyester blend. Sankhyan *et al.*^15^ reported pressure drops in the range 40–55 Pa for an air speed of $8\;{\text{cm}\;s}^{-1}$, which gives impedances in the range 5– $7\;{\text{Pa}\;s\;\text{cm}}^{-1}$.

Hancock *et al.*^19^ estimated that the American N95 standard for breathability requires a maximum impedance of around $30\;{\text{Pa}\;s\;\text{cm}}^{-1}$, four times our fabric's value. So, we conclude that the impedance of our imaged fabric is well within the range of values that are easy to breathe through.

#### 1. Model for the Darcy's law permeability

Van den Brekel^8^ used the Kozeny, or Kozeny–Carman, model for *k*. This model was developed for beds composed of packed spheres. Although van den Brekel proposed that the vast majority of the flow is through inter-yarn pores, these pores do not resemble the gaps between the sphere in beds of packed spheres. They are channels partially obstructed by stray fibers. Thus, we model *k* of our fabric by Poiseuille flow in cylinders of effective diameter *d_EFF_* that occupy an area fraction *ε_by_* of the fabric. This gives

$$k∼\frac{\varepsilon_{by}d_{\mathrm{EFF}}^{2}}{32}.$$ (7)

We estimate the effective free diameter to be in between a fiber diameter and a yarn diameter, $d_{\mathrm{EFF}}∼$ 50 *μ*m, while the area fraction of inter-yarn pores $\varepsilon_{by}∼0.1$. These values give k∼ 8 *μ*m^2^—the same order of magnitude as our measured value. Given the numerous approximations—we estimate the channel size and pore fraction, the channels are too short for a fully developed Poiseuille flow, and there are fibers that cross the channels—we consider this reasonable agreement. Bourrianne *et al.*^27^ found a similar value, *k* = 12 *μ*m^2^ for a surgical mask. This is consistent with the flow being predominantly through pores tens of micrometers across, occupying about ten percent of the total area.

### C. Curvature of streamlines

The inertia of a particle only affects its motion when the streamlines are curving. For flow that is just straight ahead, the particle will just follow the flow. So, we need to characterize the curvature of the streamlines going through the fabric. We do this by determining a characteristic lengthscale for this curvature, which we call Σ.

The lengthscale Σ for the curvature of a streamline at a point on the streamline of the flow field is defined by

$$\Sigma =\frac{\vec{u}.\vec{u}}{a_{⊥}},$$ (8)

where $\vec{u}$ is the flow field at that point and $a_{⊥}$ is the magnitude of the normal component of the acceleration $\vec{a}$ along the streamline at this point. Streamlines are defined by velocities and accelerations and so one way to obtain the lengthscale is to square the velocity and divide by the acceleration.

The acceleration is that along the streamline, i.e., rate of change of streamline velocity while being advected along the streamline. The normal component is obtained by subtracting the parallel component from $\vec{a}$ as follows:

$${\vec{a}}_{⊥}=a-\hat{u}(\hat{u}.\vec{a}).$$ (9)

**FIG. 8.**
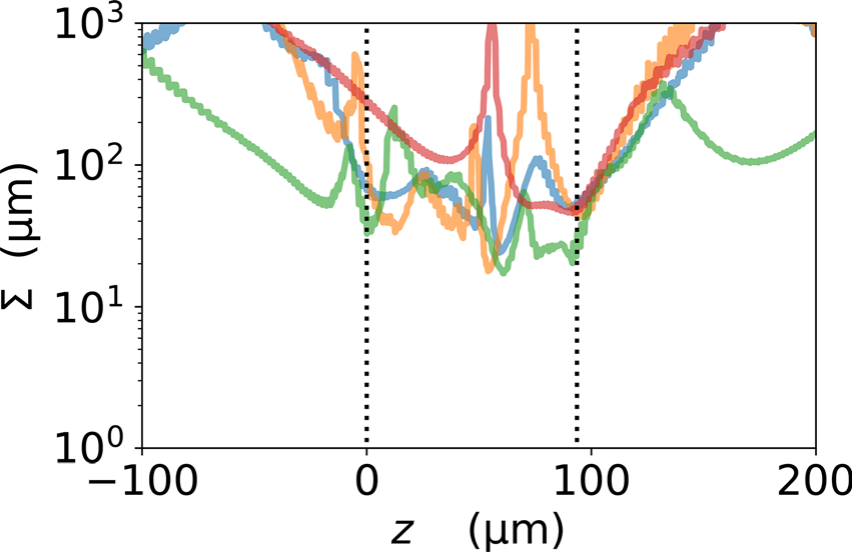
Plot of the local curvature Σ along four streamlines, as a function of their position along the flow direction *z*. The vertical dotted lines mark the start and end of the fabric, so outside of these lines we are outside the fabric. N.B. the curves are not smooth because Σ depends on the acceleration. The flow field velocity is obtained by interpolation; so, the velocity is continuous but its derivative, the acceleration, is not.

We have plotted Σ along a set of streamlines in Fig. 8. The local curvature along streamlines within the fabric varies greatly but is mostly around tens to hundreds of micrometers. This is different from the flow in a mesh of single fibers, as found in surgical masks. In surgical masks, there is only one lengthscale, that of the fiber diameter, which varies but can, for example, be around 15 *μ*m.^10^ So in non-woven filters such as surgical masks, the curvature lengthscale is expected to fall as low as around 10 *μ*m for trajectories near the surfaces of fibers.

## V. CALCULATING PARTICLE TRAJECTORIES AND COLLISIONS

In this section, we first introduce the theory for particles moving in a flowing fluid, then describe the details of our calculations.

### A. Theory for a particle in a flowing fluid

The particles are spheres of diameter *d_p_*, and feel only the Stokes drag of the surrounding air. We neglect any perturbation by the particles of the flow field, and assume that the drag force on a particle couples to its center of mass. Then Newton's Second Law for the particle becomes

$$m_{p}\frac{d\vec{v}}{dt}=-\frac{3\pi \mu d_{p}}{C}\left(\vec{v}-\vec{u}\right)$$ (10)

for a particle of mass *m_p_* and velocity $\vec{v}$ in a flow field $\vec{u}$ of fluid with viscosity *μ*. Here, *C* is the Cunningham slip correction factor.^59,60^ We consider particles with $d_{p}\geq$ 1 *μ*m (due to limited imaging resolution). In this size range, *C* is always close to one (within 15%). Therefore, we just set *C *=* *1 here.

The particles are spheres of mucus, which we assume has the mass density of water, *ρ_p_*. Then, $m_{p}=(\pi /6)d_{p}^{3}\rho_{p}$, and Eq. (10) becomes

$$\frac{d\vec{v}}{dt}=-\frac{18\mu }{\rho_{p}d_{p}^{2}C}\left(\vec{v}-\vec{u}\right)=-\frac{\left(\vec{v}-\vec{u}\right)}{t_{I}},$$ (11)

where we have introduced $t_{I}=\rho_{p}d_{p}^{2}C/(18\mu )$: the timescale for viscous drag to accelerate the particle.

#### 1. The Stokes number

When integrating Eq. (11), if the timescale *t_I_* is short then the particle closely follows (the streamlines of) the fluid flow; so when the fluid flows around an obstacle, the particle follows the fluid. However, if *t_I_* is large, then when the fluid flow changes direction the particle's inertia results in it carrying on and moving in the direction of the fluid before it changed direction. This inertial effect can result in a particle colliding with an obstacle, although the fluid flows around it, and is the cause of inertial filtration.^6^ The short and long timescales *t_I_* are relative to the timescale for the change of direction of the fluid flow, and the ratio of these two timescales defines a dimensionless number: the Stokes number.

The ratio of the timescale *t_I_* to the timescale for fluid flow to change direction as it goes around an obstacle of size *L_O_* defines the Stokes number, i.e.,

$$\text{St}(d_{p},L_{O},U)=\frac{t_{I}}{L_{O}/U},$$ (12)

where we use the Darcy speed *U*. Then,

$$\text{St}(d_{p},L_{O},U)=\frac{\rho_{p}d_{p}^{2}UC}{18\mu L_{O}}∼\frac{3.08\times 10^{6}}{m^{2}s^{-1}}\frac{d_{p}^{2}}{L_{O}}U.$$ (13)

The parameter values in Table II were used. For St≪1, viscous forces dominate inertia and the particle follows streamlines faithfully. However, for St≫1, inertia dominates and the particle's trajectory will strongly deviate from streamlines. As the streamlines go around obstacles, deviating from streamlines can result in the particle colliding with an obstacle and being filtered out. This is inertial filtration.

**FIG. 9.**
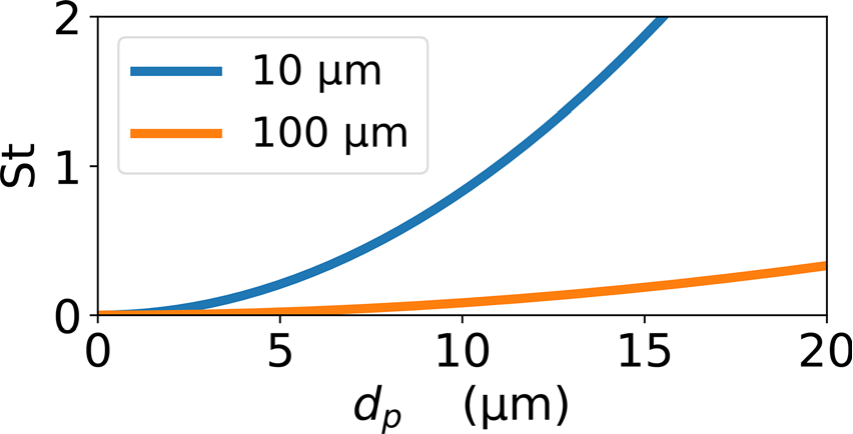
Plot of the Stokes number as a function of particle diameter *d_p_*, using Eq. (13). The blue and orange curves are for obstacle sizes *L_O_* = 10  and 100 *μ*m, respectively. The flow speed is set to $U=2.7\;{\text{cm}\;s}^{-1}$.

The Stokes number depends on the flow speed, and on both the size of the particle and of the obstacle the flow is going around. Figure 9 shows the Stokes number as a function of particle diameter, for particles in flow fields curving over lengthscales of 10 and 100 *μ*m. Note that for flow fields curving over a distance 10 *μ*m, a Stokes number of one is only reached for particles greater than 10 *μ*m in diameter. So, our fabric where the curvature Σ is mainly at least tens of micrometers (see Fig. 8) is expected to show little inertial filtration of any particle around 10 *μ*m or smaller in diameter.

### B. Evaluation of filtration using our lattice Boltzmann flow field

The filtration efficiency is estimated from the fraction of particles that collide with the fabric. We calculate the trajectories of *N_samp_* particles that start in a uniform grid that occupies the central quarter of the area in the white rectangle in Fig. 2. This area in the white rectangle is two lattice constants of the fabric across along both the *x* and *y* axes, and so the area the particles start from fills one unit cell of the fabric lattice. Our filtration efficiency should therefore be a good representation of the average filtration efficiency of a large area of fabric. Once we have computed the trajectories of the *N_samp_* particles and determined which ones collide with the fabric, the filtration efficiency is computed as follows:

$$\text{Filtration}\;\text{efficiency}=\frac{\sum_{i}^{\text{coll}}v_{zi}}{\sum_{i}^{\text{coll}}v_{zi}+\sum_{i}^{\text{pen}}v_{zi}},$$ (14)

where the sum with superscript “coll” is over all particles that collided with a fiber voxel, and the sum with superscript “pen” is over all particles that pass through the fabric without colliding. *v_zi_* is the *z* component of the velocity of particle *i* at the starting point of its trajectory. Note that as we are interested in the fraction of particles filtered, each particle is weighted by the local velocity. We assume the particle concentration is uniform in the air, so regions where the air is flowing faster contribute more than where the regions are flowing more slowly.

See the Appendix for further details of how we compute trajectories, and the condition for collisions. All calculations are for flow at the speed $U=2.7\;{\text{cm}\;s}^{-1}$, corresponding to breathing under moderate exertion (see Table II).

## VI. RESULTS FOR PARTICLE FILTRATION

**FIG. 10.**
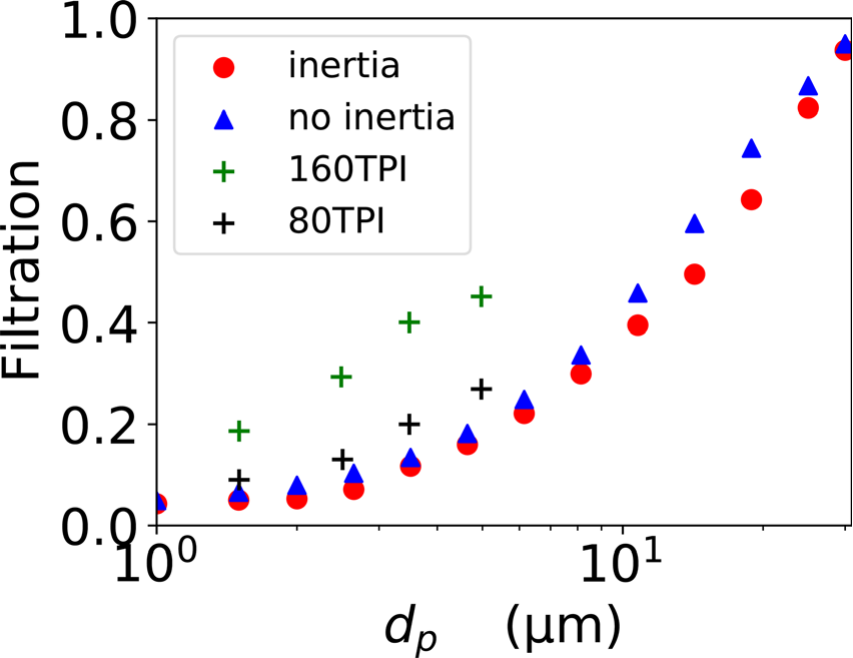
Plot of the fraction of particles filtered, as a function of their diameter *d_p_*. This is in air with flow speed $U=2.7\;{\text{cm}\;s}^{-1}$. The red circles are with the inertia of a particle with the mass density of water and the blue triangles are without inertia. They are each averages over $N_{\text{samp}}=1600$ particle trajectories. The green and black pluses are measurements of Konda *et al.*^13^ [obtained from Fig. 2(b)^61^]. These measurements are for a pressure drop across the fabric of 10 Pa, whereas at our value of *U*, the estimated pressure drop is 19 Pa. The impedances measured by Konda *et al.*^13^ are lower than our value ( $7.1\;{\text{Pa}\;s\;\text{cm}}^{-1}$), they find values of $1.3\;{\text{Pa}\;s\;\text{cm}}^{-1}$ for 80 TPI, and $4.2\;{\text{Pa}\;s\;\text{cm}}^{-1}$ for 160 TPI. Thus, especially for the 80 TPI fabric, although their pressure drop is lower, the air speed is higher.

In Fig. 10, we have plotted results for the fraction of particles that collide with a fiber and are filtered out, as a function of the diameter of the particle. These are the red data points. We see that the efficiency is less than 10% for micrometer-sized particles, and although it increases with increasing size we are still filtering less than half of the particles at a diameter of 10 *μ*m. We breathe out droplets with a wide range of sizes but the peak of this distribution is around one micrometer.^23^ We predict that the fabric we have imaged is very poor at filtering out droplets of this size. Note that we could only image approximately half of one cotton fabric layer; presumably the filtration efficiency of the full layer is higher.

Both Konda *et al.*^12,13^ and Duncan *et al.*^14^ have measured the filtration efficiency of woven fabrics, for particles up to five micrometers. Both groups find a large variability in filtration efficiency from one material to another, with filtration efficiencies in the range less than 10% to almost 100%, for particles with diameters of a few micrometers. Sankhyan *et al.*^15^ found comparable filtration efficiencies to Konda *et al.* and Duncan *et al.* They also found that the fabric masks were systematically less good at filtering than non-woven surgical masks.

Two data sets from Konda *et al.*^13^ are plotted in Fig. 10. Konda *et al.*^12,13^ found that the filtration efficiency of a fabric increased with its TPI. In Fig. 10, we see that they found that the filtration efficiency for a 160 TPI cotton/polyester fabric is higher than for 80 TPI cottons. We estimate that our fabric's TPI is 186. Our efficiencies are lower than those measured by Konda *et al.*^13^ but the slope is very similar. At a diameter of 1.5 *μ*m, we find an efficiency of 5%, whereas Konda *et al.*^13^ found efficiencies of 9% and 19% for TPIs of 80 and 180, respectively. Our model makes a number of approximations—flow field on a 1.8 *μ*m lattice, possible changes in the fibers and yarns due to immersion in the solvent, coupling at center of mass, etc.—so our estimated efficiencies are likely only accurate to within a factor of two in either direction. With this estimate of uncertainty in our calculation, we estimate an efficiency in the range 2.5%–10%. Thus, within our large uncertainties our results are essentially consistent with the measurements.

### A. Inertia can cause collisions to be avoided and so reduce filtration efficiency

In order to understand the role of inertia in filtration by woven fabrics, we calculated the filtration efficiency without inertia. In other words, the Stokes number is zero and the particles follow the streamlines perfectly. The results are shown as blue triangles in Fig. 10, and are for pure interception filtration. If we compare those points with the red points, which correspond to the case with inertia, we see that the difference is small. Inertia has a small effect and the filtration is mainly through interception.

**FIG. 11.**
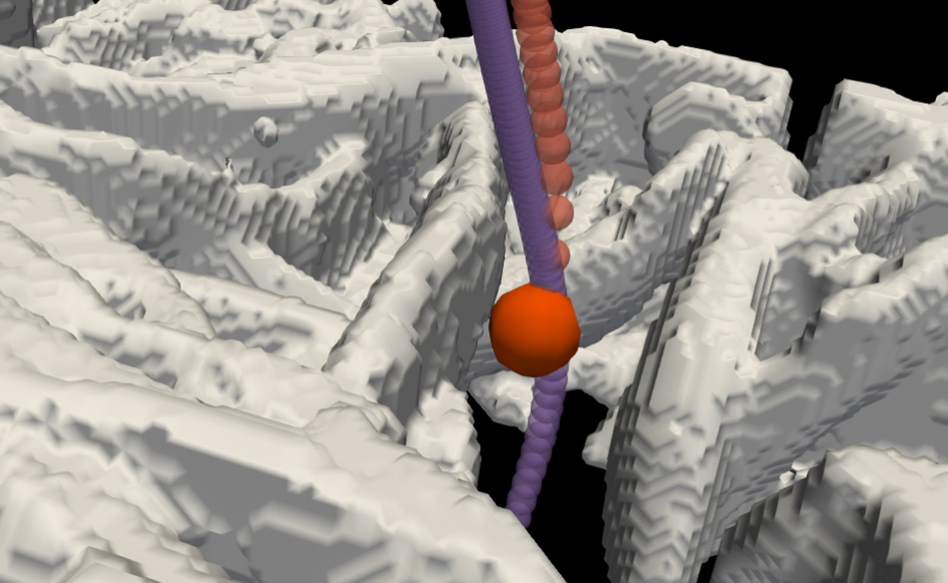
A pair of trajectories with and without inertia that start at the same point. This is for a particle of diameter 20 *μ*m. The fabric is shown in white, and trajectories with and without inertia are traced out by purple and by orange spheres, respectively. The sphere at the collision point is shown at the true particle size, others along the path are smaller, for clarity. Note that with inertia, the particle penetrates the fabric, while without it, the particle collides at the point shown by the large orange sphere. Here, inertia carries the particle a little farther out from the side of the inter-yarn pore, avoiding a collision. Image produced with ParaView.^55^

However, the difference is that the effect of inertia is to slightly decrease filtration. We have found that the effect of inertia can be to cause a collision that occurs without inertia to be avoided, see Fig. 11. There, we have plotted two trajectories with the same starting point but with inertia (purple) and without inertia (orange). The particle with inertia penetrates the fabric, while without inertia it collides with the side of the inter-yarn pore and is filtered out. Inertia carries a particle closer to the center of an inter-yarn pore where it is further from the sides and so escapes colliding with these walls.

In the standard picture of filtration of particles from air, the effect of inertia is always to increase the filtration efficiency.^6^ In that standard picture, deviations of particle trajectories from streamlines due to inertia always increase the probability of a collision. This is not what we have found, see Fig. 11. Here and in Robinson *et al.,*^10^ we find that at small Stokes numbers the situation can be more complex and subtle. Inertia at small Stokes number can make filtration a little less efficient. However, at large Stokes number, we indeed find that inertia increases the filtration efficiency.

The zero Stokes number (i.e., zero inertia) limit, often called interception filtration,^6^ corresponds to the limit in which the air speed U→0, as *U *=* *0 gives a Stokes number of zero. Thus, we have shown that reducing *U* from a speed characteristic of moderate exercise to zero has little effect on the filtration efficiency. Filtration by our fabric is almost independent of *U* or, equivalently, of the pressure drop across the fabric. This is in agreement with the findings of Konda *et al.*^13^ who found that filtration did not vary significantly when they varied the pressure drop across the sample.

## VII. FILTRATION VIA PARTICLES DIFFUSING INTO CONTACT

The filtration of particles of order 100 nm is typically dominated by the diffusion of these particles onto the surfaces of the filter.^6^ The nanoparticles then stick and are filtered out. With a flow field based on imaging at 1.8 *μ*m resolution, we are unable to be quantitative about the filtration efficiency for particles in this size range. However, we are able to argue that the efficiency of filtration by diffusion should be low. The argument is as follows:

For our fabric, almost all air flows through inter-yarn pores ∼50 *μ*m across. So, filtration by diffusion depends on particle diffusion across the flowing air stream in contact with the sides of the inter-yarn pore, during the short time the particle is being advected through the fabric. Thus, the filtration efficiency is determined by the ratio of the diffusive time *t_DX_* to the advection time *t_A_*. *t_DX_* is the time taken to diffuse across (i.e., in *xy* plane) an inter-yarn pore. *t_A_* is the time taken for air to flow through the pore.

The ratio of diffusive to flow timescales defines a Péclet number. Here, the Péclet number is

$$\text{Pe}=\frac{t_{DX}}{t_{A}}.$$ (15)

For a particle 100 nm in diameter, the Stokes–Einstein relation gives $D=kT/(3\pi \mu d_{p})∼240\;\mu m^{2}s^{-1}$, and so for a distance of 50 *μ*m, $t_{DX}∼(50^{2})/80–10\;s$. The advection timescale is just the time taken for air to flow through the fabric $t_{A}∼100\;\mu m/2.7\;{\text{cm}\;s}^{-1}∼4\;\text{ms}$. Thus,

Pe∼3000. (16)

As Pe≫1, particles with $d_{p}=100\;\text{nm}$ are carried through the fabric much faster than they can diffuse across the inter-yarn pores, and we expect the efficiency of filtration by diffusion to be very low. Note that for larger particles, *D* is smaller; so, filtration by diffusion is even less efficient.

Our prediction that filtration via diffusion should be very inefficient is consistent with a number of experimental studies.^13,14,16,62^ These studies all found that woven fabrics are poor at filtering particles much less than a micrometer in diameter, which is the size range where particle diffusion is fastest. Here, poor filtration means typically less than 50%, and in some cases much less. For diameters less than a micrometer, woven fabrics are typically poorer filters than the non-woven materials used in surgical masks. For the non-woven materials in surgical masks, at diameters around 100 nm, the efficiency increases as the diameter decreases, due to diffusion becoming increasingly important as the diameter increases.^6,10,13,14,16,30,62^ This increase is also seen in woven fabrics^13,14,16,62^ but is mostly weaker for woven than for non-woven materials.

## VIII. CONCLUSION

Measurements of the filtration efficiency of woven fabrics have consistently shown poorer filtration efficiency than for the non-woven materials used in surgical masks or other air filters.^13,14,16,19,62^ This is for the filtration of particles both smaller than and larger than a micrometer, and for a range of different fabrics of different TPIs and materials (cotton, polyester, etc.). For the first time, we have the complete flow field (at a resolution of 1.8 *μ*m) inside the fabric, and we can also control the inertia of the particles, so we can see why the efficiency is so low. The efficiency is low because essentially all the air flows through relatively large (tens of micrometers) inter-yarn pores, which are only obstructed by a few stray fibers, see Fig. 5. Particles just follow the air through these gaps and so few are filtered out.

Inter-yarn pores will vary in size from one woven fabric to another, for example they should be smaller when the TPI is larger. Some data suggest that fabrics with higher TPIs are better filters,^13^ possibly because the inter-yarn pores are smaller. However, all woven fabrics are made of yarn and so all will have inter-yarn pores. This, together with the multiple experimental studies reporting poor filtration efficiency,^13,14,16,19,62^ suggests that poor filtration is generic, because as we have seen particles are just carried through the relatively large inter-yarn gaps. These gaps are an order of magnitude greater in size than typical fiber spacings in the non-woven material in surgical masks.^10,11^

We estimate that the filtration efficiency of our imaged fabric is in the range 2.5%–10%. This is for particles of diameter 1.5 *μ*m, which is around the most probable size for droplets exhaled while speaking.^23^ Thus, this is the most probable droplet size for source control. To protect the mask wearer, the mask must filter droplets that have evaporated in the surrounding air. Because the ?ltration ef?ciency decreases with decreasing particle size, the filtration efficiency will be even lower for droplets once they have^23,29,30^ entered room air, and evaporation has reduced their diameter by a factor of 2 to 3.^23,30^ Our filtration efficiency is for approximately half a layer of woven fabric with an estimated TPI of 186. Konda *et al.*^13^ found filtration efficiencies of 9% and 18% for (complete single layers of) woven fabrics of 80 and 160 TPI. Sankhyan *et al.*^15^ also found similar values.

### A. It may be impossible to make good filters from woven fabrics

The efficiency of filtration by fabrics can be improved by using multiple layers.^15^ However, both multiple layers and higher TPI lead to higher impedance to air flow. Making a practical air filter always involves a trade-off between maximizing filtration and keeping the impedance (pressure drop) low enough to be acceptable to the user. In other words, the

$$\text{figure}\;\text{of}\;\text{merit}\;\text{for}\;a\;\text{filter}=\frac{-\text{ln}\;[1-\text{Fraction}\;\text{Filtered}]}{I}.$$ (17)

Our estimated impedance of $I=7.1\;{\text{Pa}\;s\;\text{cm}}^{-1}$ is low in the sense that it is approximately one-quarter the maximum impedance allowed by the American N95 standard.^19^ However, due to the very low filtration efficiency, the value of the figure of merit is low for our fabric. Taking our 5% filtration efficiency for 1.5 *μ*m, our estimated figure of merit is $0.007\;{\text{cm}\;\text{Pa}}^{-1}\;s^{-1}$. Achieving 95% filtration at the maximum impedance allowed by an N95 mask requires a figure of merit of $0.1\;{\text{cm}\;\text{Pa}}^{-1}\;s^{-1}$, more than ten times the value for our cotton fabric. Here, we used the estimated maximum impendance of the N95 standard of $30\;{\text{Pa}\;s\;\text{cm}}^{-1}$ of Hancock *et al.*^19^ It may be that it is impossible or almost impossible to make good filters from fabrics, because their figures of merit for filtration are too low.

### B. The effect of particle inertia on filtration

We find that for our woven fabric, filtration is mostly due to interception over the size range from one to a few tens of micrometers. In other words, filtration is due to particles that largely follow the streamlines but collide with cotton fibers due to the particle's size.^6^ Note that filtration is only weakly affected by setting the inertia of particles to zero, compare the blue and red points in Fig. 10. Surprisingly, over this size range, the effect of inertia is to decrease filtration efficiencies, although the effect is small. Modest amounts of inertia decrease the filtration efficiency by pushing more particle trajectories away from collisions with fibers, than they do trajectories toward collisions. Very large amounts of inertia (for example, due to a sneeze greatly increasing *U*) will increase the efficiency due to most of the fabric area being occupied by yarns.

The non-woven filters in surgical masks and respirators (such as the European standard FFP and American standard N95 respirators) force the air around single fibers of typical size around 5 *μ*m.^11^ This smaller lengthscale for the curvature of streamlines in surgical masks brings inertial filtration into play for droplets around a few micrometers in diameter.^10^ This makes inertial filtration much more effective for surgical masks and respirators than for woven fabrics, for particles one or a few micrometers in diameter.

### C. Limitations of the present work, and future work

We have simulated the flow field through one sample of woven fabric at a resolution of 1.8 *μ*m, and used this to understand the observed poor filtration performance. Future work could look at different fabrics, with different TPIs, and go to higher resolutions, as well as compare with the materials used in surgical masks.^20,21^ Higher resolution images will improve the estimation of filtration of smaller particles in particular, as this is likely to be sensitive to yarn/fiber roughness of lengthscales of a micrometer and smaller.

## Data Availability

The data and computer code that support the findings of this study are openly available in Zenodo at http://doi.org/10.5281/zenodo.5552357, Ref. 63.

## APPENDIX: COMPUTATIONAL DETAILS FOR INTEGRATING PARTICLE TRAJECTORIES

Each particle trajectory is obtained by starting the particle at *z *=* *5 in LB units, and at *x* and *y* coordinates on a square grid in the central quarter of the box, i.e., from $n_{x}/4$ to $3n_{x}/4$ along the *x* axis and from $n_{y}/4$ to $3n_{y}/4$ along the *y* axis. We varied the starting region for the trajectories to observe the dependence of efficiency on the starting region, and the efficiency varied by amounts around 10%. The particle starts with the same velocity as the local flow velocity. Weighting the trajectories by their initial velocities using Eq. (14) makes a difference of approximately twenty percent for our box with 200 LB lattice spacings in front of the fabric. It makes more of a difference for shorter boxes along *z*, hence our box size is a trade-off between accuracy and computational cost.

A fraction of order 20% of the trajectories leave the box at the sides. These are not counted in our flux calculations. Although the LB flow field has periodic boundary conditions at the sides, this does not reproduce well the true conditions in the fabric, which is not perfectly periodic in the *x* and *y* directions. In the xy-plane the simulation box cannot be larger than shown by the white box in Fig. 2. Enlarging it to the left expands the box to include the defect immediately to the left of the white box, while enlarging it along the y axis reaches the edges of the strip of fabric imaged.

So, we have multiple sources of uncertainties, each ten or a few tens of percent. Plus, we only couple the particle to the fluid flow at the particle's center of mass, and are using a flow field with spatial resolution larger than the smallest particles we consider. Considering all these sources of uncertainty, and the approximations of the model, we estimate that our results are accurate to about a factor of 2.

Each trajectory is integrated forward in time, using adaptive-step-size modified Euler integration of Eq. (11), until the particle either collides with a fiber voxel, or reaches the bottom (large *z*) edge of the simulation box. At each time step, we check for a collision. A collision occurs if the center of the particle is within a distance $\left(1/2)(d_{p}+\delta \right)$, i.e., the radius of the particle plus a correction *δ*. We estimate that the optimal value of *δ* is 0.5 in LB units. So, we use this value throughout this work.

The integration of Eq. (11) requires that we determine *t_I_* in LB units. This is done as follows, for the example of a particle with *d_p_* =5 *μ*m. First, we obtain the mean velocity in the LB flow field in a slice far from the fabric, as $U=5.8\times 10^{-7}$ in LB units. Second, we use Eq. (13) to determine that St=1.16, for lengthscale *L *=* *1.8 *μ*m and $U=2.7\;{\text{cm}\;s}^{-1}$. Then, we use Eq. (12) in LB units to obtain, with *L *=* *1 and our LB *U*, that $t_{I}=20.8\times 10^{6}$ in LB units. This value of *t_I_* reproduces the correct Stokes number for a particle 5 *μ*m in diameter. The particle then collides with any lattice site within a distance of 1.89 LB units.
